# The Vibrational and Thermodynamic Properties of CsPbI_3_ Polymorphs: An Improved Description Based on the SCAN meta-GGA Functional

**DOI:** 10.1021/acs.jpclett.1c01798

**Published:** 2021-07-12

**Authors:** Jakub Kaczkowski, Iwona Płowaś-Korus

**Affiliations:** Institute of Molecular Physics, Polish Academy of Sciences, M. Smoluchowskiego 17, 60-179 Poznań, Poland

## Abstract

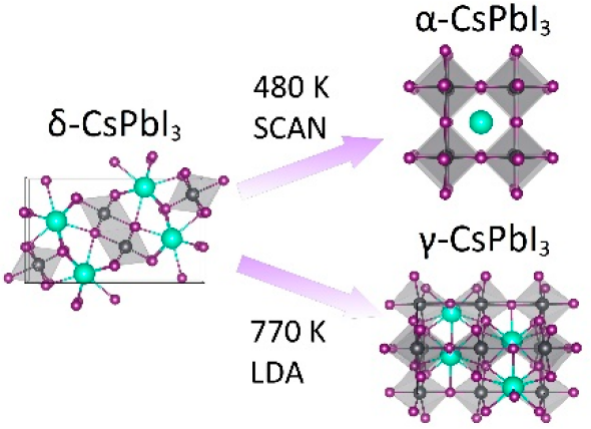

We report the vibrational and thermodynamic properties of four known CsPbI_3_ polymorphs in the framework of the density functional theory. We compare the recently introduced strongly constrained and appropriately normed (SCAN) meta-generalized gradient approximation (meta-GGA) with the local density approximation (LDA). We found that the SCAN, compared to the LDA, could explain discrepancies between theoretical and experimental results. Evaluating the Helmholtz free energy as a function of temperature, we found that within the SCAN (a) all polymorphs had negative formation enthalpies at the room temperature and (b) CsPbI_3_ underwent the phase transition from the δ- to α-phase at 480 K. This is not true for the LDA. In contrast to the previous reports based on the LDA, we did not find the ferroelectric instability in the phonon spectra of the cubic and tetragonal phases at the meta-GGA level. This result agrees with the lack of observation of the ferroelectricity in CsPbI_3_.

The halide perovskites are one of the most promising materials for photovoltaic applications.^1^ This is due to the optimal band gap for the solar cell,^1−3^ high defect tolerance,^4,5^ high absorption coefficients,^6,7^ the low binding energy of excitons,^8,9^ the high charge carrier mobility and long diffusion lengths,^10−12^ and the tunable band gap.^2,13,14^ As a result, the power conversion efficiency (PCE) of a single-junction solar cell based on these materials may increase by 19%.^1,15^ In addition, these materials also have a low cost of fabrication compared to the silicon-based devices. The first perovskite solar cells were based on hybrid organic–inorganic perovskites, which suffer from the poor thermal stability because of the presence of organic cations.^1^ To overcome this problem and to improve the stability of perovskite solar cells, the replacement of organic cations with inorganic cations Cs^+^, has been proposed.^13,16^ Cesium halide perovskites CsBX_3_ (where B = Pb, Sn, and X = Cl, Br, I) exhibit better stability than their organic analogues.

Among them, CsPbI_3_ has the most suitable band gap *E*_g_ (∼1.7 eV^3,16^) according to the Shockley-Queisser model^17^ for the solar cell applications. There are four known CsPbI_3_ polymorphs, whose crystal structures are presented in Figure 1. Unfortunately, at the normal conditions CsPbI_3_ crystallizes in a yellow nonperovskite orthorhombic structure (δ-CsPbI_3_, *Pnma* space group), which is unsuitable for applications (*E*_g_ = 2.58 eV^3^). The desired perovskite black phases of CsPbI_3_ are stable at high temperatures.^16^ Upon heating, CsPbI_3_ undergoes a phase transition from orthorhombic δ- to cubic α-phase at 583 K.^16,18,19^ Marronnier et al.^19^ found that, upon cooling, the cubic α-CsPbI_3_ converted to tetragonal (β-CsPbI_3_, *P*4/*mbm* space group) and the perovskite orthorhombic (γ-CsPbI_3_, *Pbnm* space group) phase at 510 and 325 K, respectively. In ref (20) authors demonstrated that the rapid cooling of α-phase in a dry air stabilized the perovskite γ-phase at the room temperature, whereas the slow cooling led to δ-phase.

**Figure 1:**
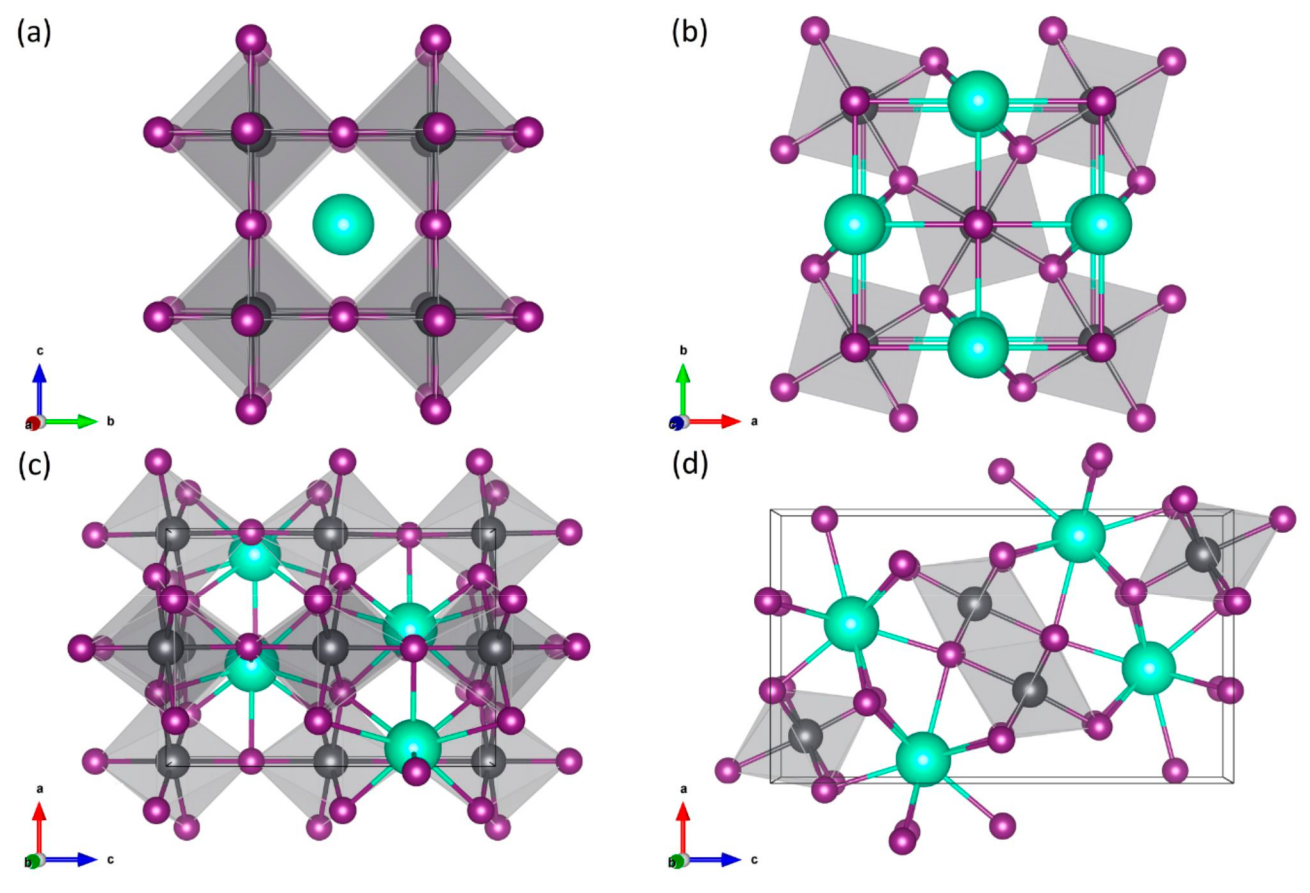
CsPbI_3_ polymorphs: (a) cubic α; (b) tetragonal β; (c) orthorhombic γ; (d) orthorhombic δ. The Cs, Pb, and I atoms are represented by bright turquoise, gray, and dark magenta spheres, respectively.

The phase transitions between halide perovskite phases can be related to the condensation of soft modes, as shown in the early experimental reports.^21,22^ On the other hand, the transition to the nonperovskite phase requires a more complex rearrangement of atoms.^23^ Thus, theoretical studies of phase transitions focus on the analysis of the phonon dispersion curves.^23−25^ Yang et al.^24^ showed that at the Brillouin zone boundary CsPbX_3_ and CsSnX_3_ in the cubic phase exhibited anharmonic phonon modes associated with the tilting of PbX_6_ and SnX_6_ octahedra. For all these modes, double-well potentials of different depths were found with a saddle point corresponding to cubic α-phase. As a result, α-phase can be considered as a dynamical average between the lower symmetry phases.^24^ Marronnier et al. investigated the lattice dynamics of all four polymorphs of CsPbI_3_.^19,26^ Excluding the γ-phase, all polymorphs exhibit Γ-point instability, which suggests the presence of ferroelectric distortion. However, the ferroelectricity has not been observed in CsPbI_3_. This is explained by the oscillation of the CsPbI_3_ along the polar soft mode between two low-symmetry structures.^26^ In addition, the phonon dispersion of the cubic CsPbI_3_ reported by different authors^24,26,27^ reveals the presence of the structural instabilities in the whole Brillouin zone. Such a situation indicates the possible presence of numerous low-symmetry perovskite phases. However, only three perovskite polymorphs were found experimentally. In all aforementioned reports the local density as well as generalized gradient approximations for the exchange–correlation were used. It is well-known that those approximations have several drawbacks like, e.g., under- (LDA) or overestimations (GGA) of the structural properties leading to over- or underestimations of the phonon frequencies, respectively.

In the present work we exploit the meta-GGA strongly constrained and appropriately normed (SCAN) functional^28^ to investigate the structural and vibrational properties of CsPbI_3_ polymorphs. The meta-GGA functionals make up an another level of the approximation for the exchange–correlation functionals. At the lowest level, the exchange–correlation energy depends only on the local electron density (the local density approximation LDA). The next level, the generalized gradient approximation (GGA), depends on the electron density and its gradient. The meta-GGA introduces the dependence on the kinetic energy density. The uniqueness of the SCAN meta-GGA lies in the fact that it is the first functional that fulfills all known constraints on the exchange–correlation functional at the semilocal level. In its design, SCAN builds correctly the kinetic energy density in the form of a dimensionless variable which recognizes all types of the orbital overlap.^29^ As a result, SCAN better than LDA and GGA recognizes and treats different electron density regions characterizing different types of chemical bonds. It was shown that compared to the LDA and GGA, the SCAN functional improves the description of structural^30,31^ and vibrational properties^31,32^ of materials with diverse bondings. In this work we show that in the case of CsPbI_3_ polymorphs the SCAN functional leads to the more accurate description of their structural and vibrational properties and, as a result, the phase transitions compared to the other XC functionals. In particular, at the meta-GGA SCAN level we did not find Γ-point instability responsible for the ferroelectric distortion. This agrees with the lack of observation of the ferroelectricity. Moreover, at the meta-GGA level the only instabilities in the cubic phase correspond to octahedral rotations. These soft modes are responsible for lowering symmetry to the orthorhombic *Pnma* and tetragonal *P*4/*mbm* phases which are the only perovskite phases observed in the case of CsPbI_3_.

We started our study from the structural optimization of all known polymorphs of CsPbI_3_ using the LDA, GGA-PBE, and meta-GGA SCAN functionals. Our results are presented in Table 1 together with the available experimental and theoretical data from previous studies.^3,19,20,33^ The lattice constants obtained from LDA and GGA are in good agreement with those determined by other authors using the same approximations.^19,33^ Compared to the experimental results,^3,19,20^ the LDA lattice constants are slightly underestimated, whereas the GGA-PBE values are overestimated, as expected. Expect the value of *b* constant in γ phase, which underestimated by nearly 8% in the LDA, the relative error in other cases is about 3% for both LDA and GGA. In contrast, the meta-GGA SCAN functional leads to the better agreement with the experimental values compared to LDA and GGA. As a result, the relative error is also reduced and do not exceed 1.8% in all cases for SCAN functional.

**Table 1:** Calculated Theoretical Lattice Parameters of CsPbI_3_ Polymorphs within Different Exchange–Correlation Functionals (LDA, GGA, and meta-GGA SCAN) along with the Available Experimental Data and Other Theoretical Results for Comparison

		LDA		GGA-PBE		SCAN
	experiment	this work	other calc	this work	other calc	this work
α-CsPbI_3_ (*Pm*3̅*m*)						
*a* [Å]	6.2965^a^	6.1405	6.149^a^	6.3864	6.40^d^	6.3026
β-CsPbI_3_ (*P*4/*mbm*)						
*a* [Å]	8.8269^a^	8.4363	8.447^a^	8.8391		8.7135
*c* [Å]	6.299^a^	6.2699	6.273^a^	6.4785		6.4132
γ-CsPbI_3_ (*Pnam*)						
*a* [Å]	8.8518;^a^ 8.8561;^b^ 8.85783^c^	8.9391	8.959^a^	9.0951	9.13^d^	9.0182
*b* [Å]	8.6198;^a^ 8.5766;^b^ 8.8637^c^	7.9465	7.928^a^	8.7308	8.66^d^	8.5224
*c* [Å]	12.5013;^a^ 12.4722;^b^ 12.4838^c^	12.2169	12.224^a^	12.6378	12.64^d^	12.5202
δ-CsPbI_3_ (*Pnma*)						
*a* [Å]	10.462;^b^ 10.450^c^	10.1634		10.8948	10.79^d^	10.6019
*b* [Å]	4.799;^b^ 4.7965^c^	4.6785		4.8808	4.89^d^	4.8185
*c* [Å]	17.765;^b^ 17.7602^c^	17.2660		18.2091	18.21^d^	17.9376

a Reference (19). Experimental results obtained for powder samples at 300, 510, and 640 K for α-, β-, and γ-phases, respectively.

b Reference (20). Powders and thin films at 293 K.

c Reference (3). Single crystals at 295 K.

d Reference (33).

Next, we would like to discuss the chemical stability of the CsPbI_3_ polymorphs versus decomposition into bulk CsI and PbI_2_.^34^ Using aforementioned exchange–correlation functionals, we calculated the formation enthalpies *E*_for_ of each CsPbI_3_ phase with respect to the cubic CsI (space group *Pm*3̅*m*) and hexagonal PbI_2_ (space group *P*3̅*m*1) compounds in the following way:
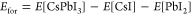
1where *E* refers to the total energy of each compound at its optimized geometry at 0 K and 0 GPa. The lower the formation enthalpy, the more stable the structure.

In Figure 2 we compare the formation enthalpies *E*_for_ of CsPbI_3_ polymorphs obtained within LDA, GGA, and meta-GGA SCAN. The qualitative picture is very similar for the all considered XC functionals and agrees with the previous theoretical reports (in the case of LDA and GGA).^20^ First of all, we observe the same behavior of formation enthalpies in CsPbI_3_ polymorphs. Second, the nonperovskite δ phase of CsPbI_3_ is the most energetically favorable one. Finally, both γ and δ phases have negative formation enthalpies for all approximations. For the δ-CsPbI_3_*E*_for_ is −150 meV/f.u., −160 meV/f.u., and −180 meV/f.u. for LDA, GGA, and SCAN, respectively, which also agrees with the recent experimental value of −180 meV/f.u.^35^ The calculated formation enthalpies of α-CsPbI_3_ are positive (180 meV/f.u. and 15 meV/f.u. for the LDA and SCAN respectively) or close to zero (−2 meV/f.u. for the GGA), which suggest that this polymorph is not chemically stable. We also found the positive value of *E*_for_ for the β-CsPbI_3_ within the LDA (38 meV/f.u.). On the other hand, for the β-CsPbI_3_, the *E*_for_ calculated within GGA and meta-GGA SCAN functionals is negative (−77 meV/f.u. and −64 meV/f.u., respectively), which indicates a stable phase.

**Figure 2:**
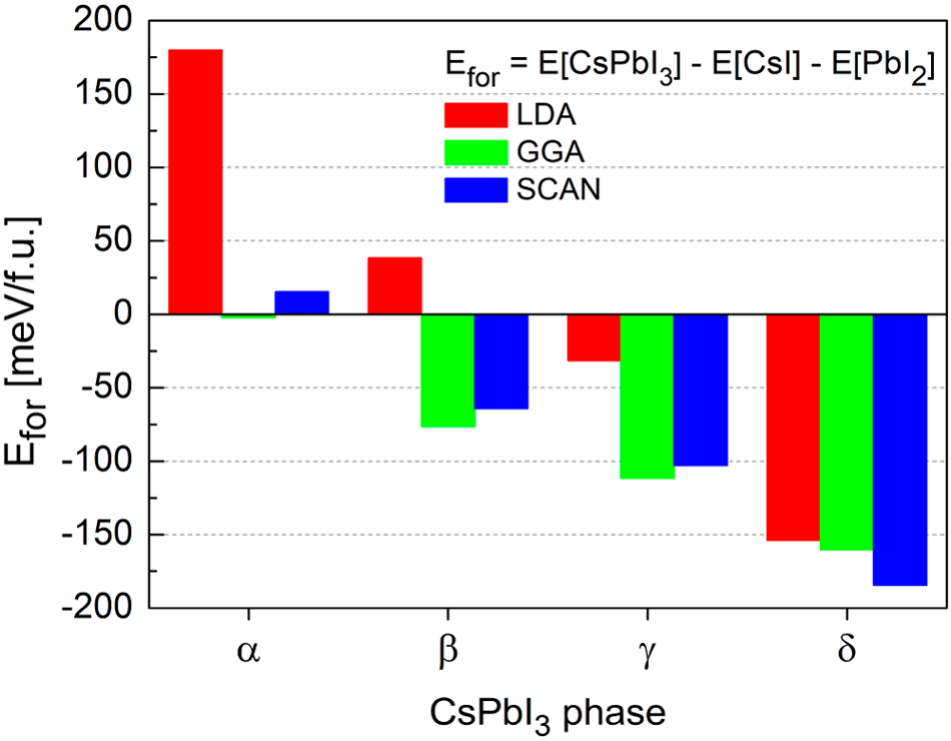
Formation enthalpies *E*_for_ of CsPbI_3_ polymorphs calculated within the LDA, GGA, and meta-GGA SCAN functionals.

The positive values of the *E*_for_ for α-CsPbI_3_ do not explain the fact that this phase was observed experimentally at the high temperatures (above 600 K^16,18,19^). However, the formation enthalpies calculated above do not take into account the contribution from the zero point motion as well as from the temperature effects. In order to include these effects we need to use the Gibbs free energy as a function of the temperature *T*, pressure *p*, and crystal volume *V* within the adiabatic approximation:
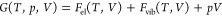
2where *F*_el_ and *F*_vib_ are electronic and vibrational contributions to Helmholtz free energy, respectively. In this work, the last term is ignored as the ambient pressure is set to 0 GPa. We also use the harmonic approximation, in which the volume dependence of *G* is neglected. As a result, the Gibbs free energy reduces to the Helmholtz free energy. The electronic contribution to the free energy *F*_el_(*T*) is given by
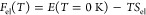
3in which the entropy contribution *TS*_el_ is omitted due to the nonmetallic character of investigated materials and *E*(*T* = 0 K) is the total energy of the system obtained from density functional calculations. The vibrational contribution to the free energy *F*_vib_(*T*) is calculated from the phonon density of states. *F*_vib_(*T* = 0 K) is the zero-point energy (ZPE). Finally, to analyze the temperature effect on the chemical stability of CsPbI_3_ polymorphs we calculated the Helmholtz free energy difference as follows:
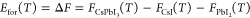
4The ZPE has some visible effects in the *E*_for_. In the case of α-CsPbI_3_, namely for the GGA the *E*_for_ decreases from −2 meV/f.u. to −16 meV/f.u., whereas for the meta-GGA SCAN the value of *E*_for_ is reduced from 15 meV/f.u. to 4 meV/f.u. For the other polymorphs the ZPE plays a minor role in the formation enthalpy *E*_for_. The experimental values of the formation enthalpies at room temperature are −29 meV/f.u. and −175 meV/f.u. for the α- and δ-CsPbI_3_, respectively.^35^ Including the vibrational free energy from the phonon calculations leads to the values of the formation enthalpies at 300 K of 228 meV/f.u., 69 meV/f.u., and −119 meV/f.u. for LDA, GGA, and SCAN, respectively. Despite the large discrepancy between the measured and calculated values of *E*_for_, only the meta-GGA SCAN leads to the negative value of the formation enthalpy at room temperature and explains its stability. For the δ-CsPbI_3_ our calculated values of *E*_for_ are −149 meV/f.u., −151 meV/f.u., and −188 meV/f.u. for the LDA, GGA, and SCAN, respectively.

In Figure 3 we presented the Helmholtz free energy difference Δ*F* of the four CsPbI_3_ polymorphs with respect to its precursors CsI and PbI_2_ as a function of temperature for the LDA (Figure 3a) and meta-GGA SCAN (Figure 3b). For the LDA the δ phase is most stable up to 770 K, which is higher than the melting point (753 K^18,20^). Above these temperatures the γ has the lowest free energy difference Δ*F*. For the cubic (α) and tetragonal (β) phases the Δ*F* is positive in the whole range of temperatures, suggesting that these phases will not form. However, the above results do not agree with the experimental observation which confirm that the cubic phase is the high temperature phase of CsPbI_3_^16,18,19^ and the tetragonal phase has also been synthesized.^15^ Despite some improvement at the GGA level, the results for both the LDA and GGA qualitatively looks similar, so we omitted the latter in the figure. Here, we only mention that within the GGA the cubic and tetragonal phases have negative formation energies up to 145 and 880 K, respectively, and the temperature of the phase transition from δ phase to γ phase is 310 K (for details see the Supporting Information, Figure S1). In Figure 2b we present the results obtained within the meta-GGA SCAN. Here, the free energy difference Δ*F* is negative for the all CsPbI_3_ polymorphs in the whole range of temperatures (with small exception for the α phase at the very low temperatures). Once again, the δ phase is the most stable polymorph. The meta-GGA results indicate the phase transition from δ to α above 480 K. Moreover, for the β and γ phases the Δ*F* is lower than for the δ above 690 K. Both values are lower than the experimental value of the melting point which is 753 K.^18,20^ The calculated temperature of the phase transition from orthorhombic to cubic is much lower than those reported experimentally (above 600 K^16,18,19^). This discrepancy could arise from the fact that we used the harmonic approximation. Further improvement could be obtained within the quasi-harmonic approximation which requires the phonon spectra of the expanded and compressed structures around the equilibrium volume. Admittedly, such calculations are computationally expensive.

**Figure 3:**
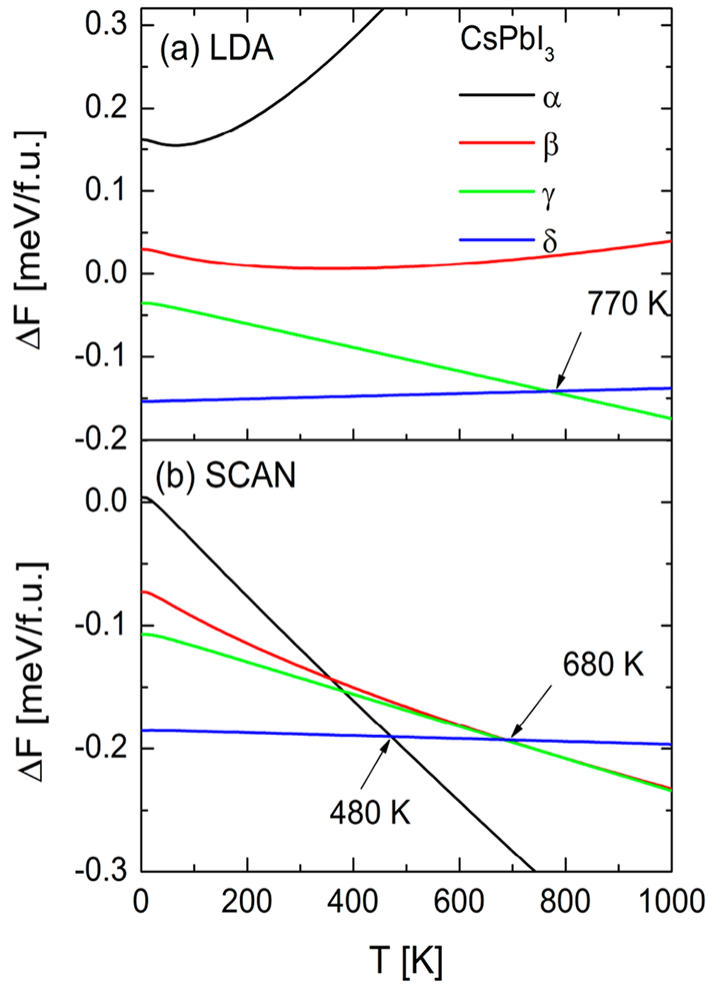
Helmholtz free energy difference for CsPbI_3_ polymorphs with respect to the CsI and PbI_2_ calculated within LDA (Figure 3a) and meta-GGA SCAN (Figure 3b).

In Figures 4–7 we present the calculated phonon dispersion relations along with the atomic-resolved phonon density of states for the four known CsPbI_3_ polymorphs obtained within the local density approximation (Figures 4a–7a) and meta-GGA SCAN (Figures 4b–7b) at their equilibrium volumes. Due to the similarities between the LDA and GGA we do not show the results for the latter here (see Figures S2–S5 in the Supporting Information).

**Figure 4:**
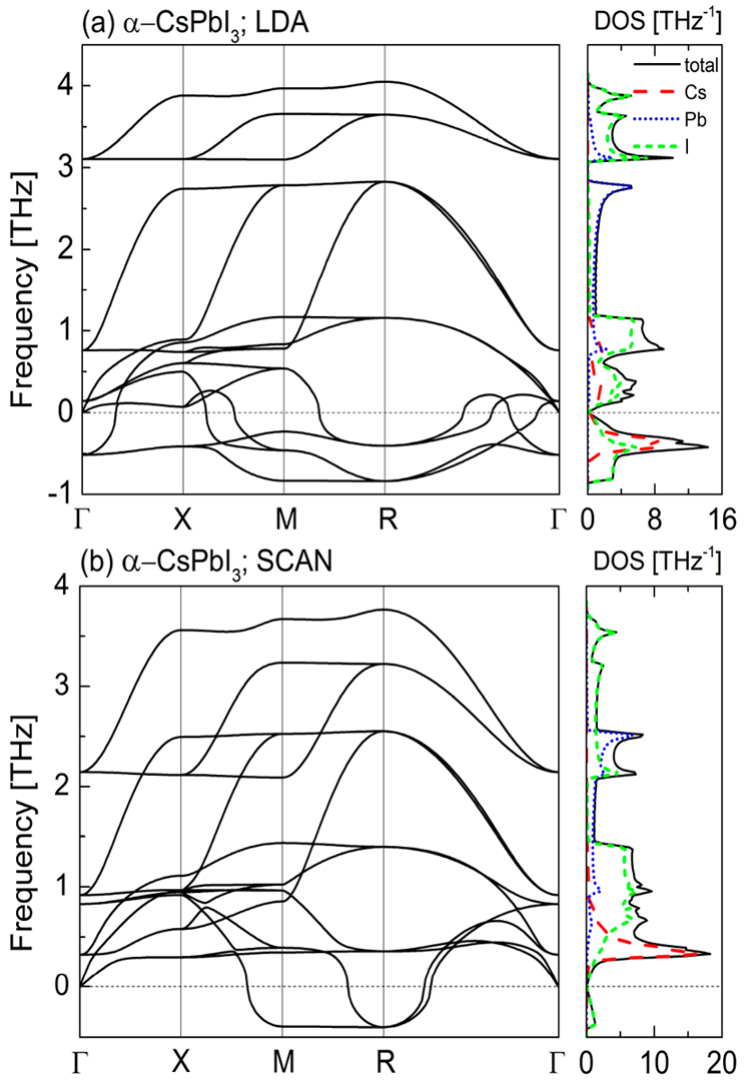
Phonon dispersion curves and the density of states for α-CsPbI_3_ calculated within (a) LDA and (b) meta-GGA SCAN.

**Figure 5:**
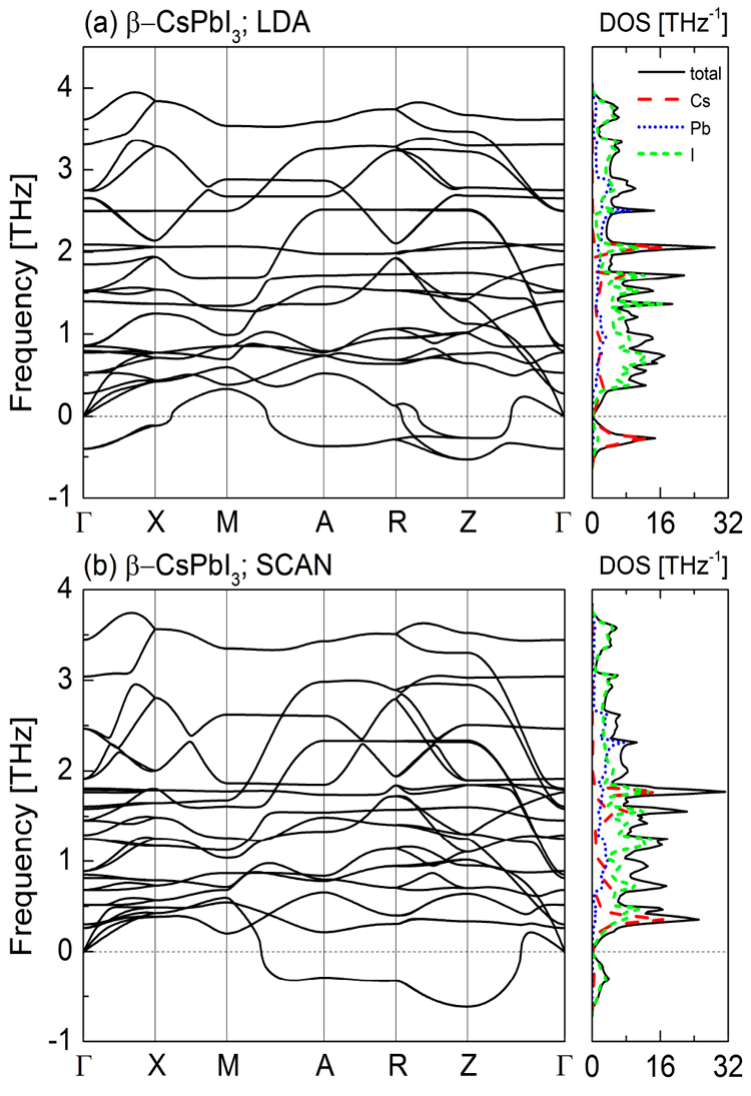
Phonon dispersion curves and the density of states for β-CsPbI_3_ calculated within (a) LDA and (b) meta-GGA SCAN.

**Figure 6:**
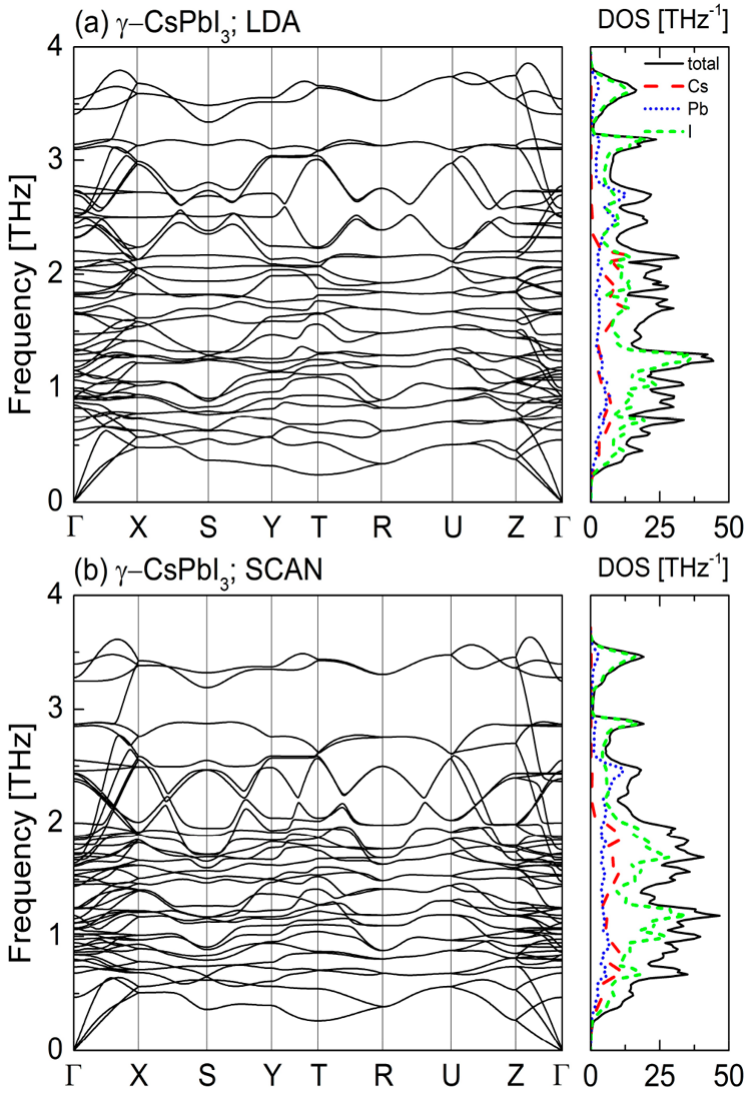
Phonon dispersion curves and the density of states for γ-CsPbI_3_ calculated within (a) LDA and (b) meta-GGA SCAN.

**Figure 7:**
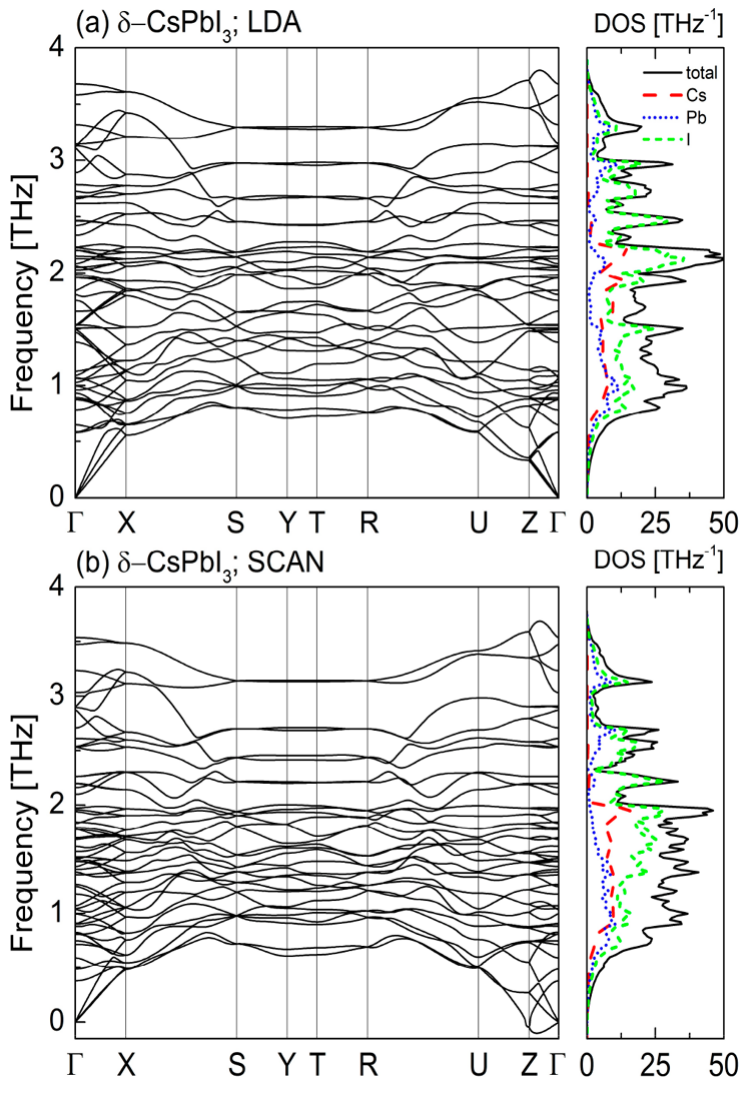
Phonon dispersion curves and the density of states for δ-CsPbI_3_ calculated within (a) LDA and (b) meta-GGA SCAN.

For the α (Figure 4) and β (Figure 5) phases both approximations reveal the presence of soft modes, indicating that these CsPbI_3_ polymorphs are dynamically unstable at *T* = 0 K. For the orthorhombic δ- and γ-phases the phonon dispersion curves obtained within the LDA (Figures 6a and 7a, respectively) do not contain any soft modes. This indicates their dynamical stability. We also did not find any instabilities in the phonon band structure of the γ-CsPbI_3_ calculated within the SCAN functional (Figure 6b). However, for the phonon dispersions of the δ-CsPbI_3_ (Figure 7b) calculated with the same functional we found small instabilities along Z−Γ direction, which could be a numerical artifact (see Figure S11 in the Supporting Information). To avoid it, the plane-wave cut off energy should be increased^25^ but this would result in the increase of computational cost.

At the beginning we look thoroughly at the structural instabilities of the α-CsPbI_3_. The analysis of unstable modes of the cubic phase is a useful and well-established method for understanding the possible phase transitions in perovskites.^36,37^ For the LDA, the imaginary frequencies are observed throughout the whole Brillouin zone including more than one instabilities at the M- and R-points, whereas for the meta-GGA SCAN there are no polar (at the Γ-point) and octahedral rotation (at the X-point) instabilities. However, for the SCAN functional there are instabilities at the M and R points but only one for each of these points, which correspond to the octahedral rotations. This is clearly visible from the phonon density of states where only iodine atoms contribute to the soft modes. The Γ-point instability observed only within the LDA is associated with a polar distortion, and its condensation corresponds to a transition to a ferroelectric phase. However, no such transition has been observed in CsPbI_3_. From the comparison of the phonon density of states within the two approximations we see that this polar distortion is associated mainly with the movement of the Cs atoms. Marronnier et al.^19,26^ showed that the ferroelectric instability of α and β phases indicated that those structures were not the true energy minima but rather a metastable phase. In order to obtain the structure with the minimum energy authors distorted the considered structures along the Γ-point soft mode. Their new structures were the true energy minima whereas the previously obtained structures were saddle points of the double-well potential with the energy higher only 7.3 meV (3.1 meV) for α-CsPbI_3_ (β-CsPbI_3_).^19,26^ As we show later in this work, the meta-GGA SCAN leads to the true energy minimum for both polymorphs. The LDA, with instabilities in the whole Brillouin zone, indicates a large variety of possible structures. On the other hand, within the SCAN functional there are only two instabilities, i.e., at the M and R points, respectively. The stabilization of the alone M-point soft mode leads to the structure with the *P*4/*mbm* symmetry whereas the stabilization of both soft modes at the M- and R-points leads to the *Pnma* symmetry. Interestingly, these phase are the only perovskite structures of CsPbI_3_ observed experimentally so far. The soft mode at the M-point is associated with the PbI_6_ octahedron rotation around the *z*-axis. As a result, the lattice constant increases along x- and *y*-axis about √2a_*Pm*3̅*m*_ whereas the along *z*-axis remains almost unchanged. The β-phase within the both approximation does not has the M-point instability in the phonon band structure. Similar to the cubic phase, the Γ-point instability is still present within the LDA but not within the SCAN.

Our first-principles calculations within the harmonic approximation show that the results obtained within the SCAN functional much better reflects the phase behavior and properties of CsPbI_3_ reported in many experimental works^3,15,16,18−20,35^ than previously used LDA and GGA.^19,24,26,27,33,34^ To shed light on the difference between the results obtained within LDA and SCAN functionals, we investigated the phonon dispersions at different volumes of the α-CsPbI_3_ as well as the electronic structure of this phase within both functionals. The former is connected with the fact that exchange–correlation functionals usually under- or overestimate the values of structural parameters whereas the latter is due to the fact that the SCAN functional provides a better description of chemical bonds than the LDA and GGA functionals.

First, we analyzed the effect of volume change. From Table 1 we see that the lattice constant of the α-CsPbI_3_ within the LDA is underestimated about 2.5% compared to both experimental and SCAN results. For this reason we calculated the phonon spectrum of the α-phase for the SCAN lattice parameter within the LDA. In addition, we also calculated the phonon spectrum of that phase within the SCAN functional for the LDA lattice constant. The results are presented in Figure 8. We see that in the case of the cubic CsPbI_3_ the change of the structural parameters do not change qualitatively the phonon band structure. For both volumes we did not find the ferroelectric instability within the meta-GGA SCAN functional. The instability is present within the LDA though. Similar to Marronnier et al.,^19,26^ we distorted structure along the Γ-point soft mode; namely we moved the Cs and I atoms in the opposite direction. The results are presented in Figure 9. We see that at the meta-GGA SCAN the obtained structure is the true energy minimum (as should be expected from the lack of the instability). The more important is the effect of the volume within the LDA. For the both considered volumes we observed the double-well potential behavior. However, for the LDA volume the height of the barrier is 5.1 meV (for comparison 7.3 meV in ref (26)), whereas for the SCAN volume (which is close to the experimental one) this value increases to 31.1 meV. Next, we performed similar analysis for the β-CsPbI_3_. The results are presented in the Supporting Information, namely the results of the phonon dispersions (Figure S6) and the energy as a function of distortion along the Γ-point soft mode (Figure S7). Interestingly, the phonon dispersion calculated within the SCAN for the LDA volume (Figure S6a) shows only one instability at the Z point. This may suggest that further compression would stabilize the β phase. On the other hand, the LDA results for the SCAN volume indicate the instabilities in the whole Brillouin zone. Once again, we did not find the ferroelectric instability within the SCAN for both volumes. Distorting atoms along the Γ-point soft mode at the LDA (Figure S3), leads to the values of the energy barrier of 1.9 meV (3.1 meV in ref (19)) and 5.9 meV for the LDA and SCAN volumes, respectively.

**Figure 8:**
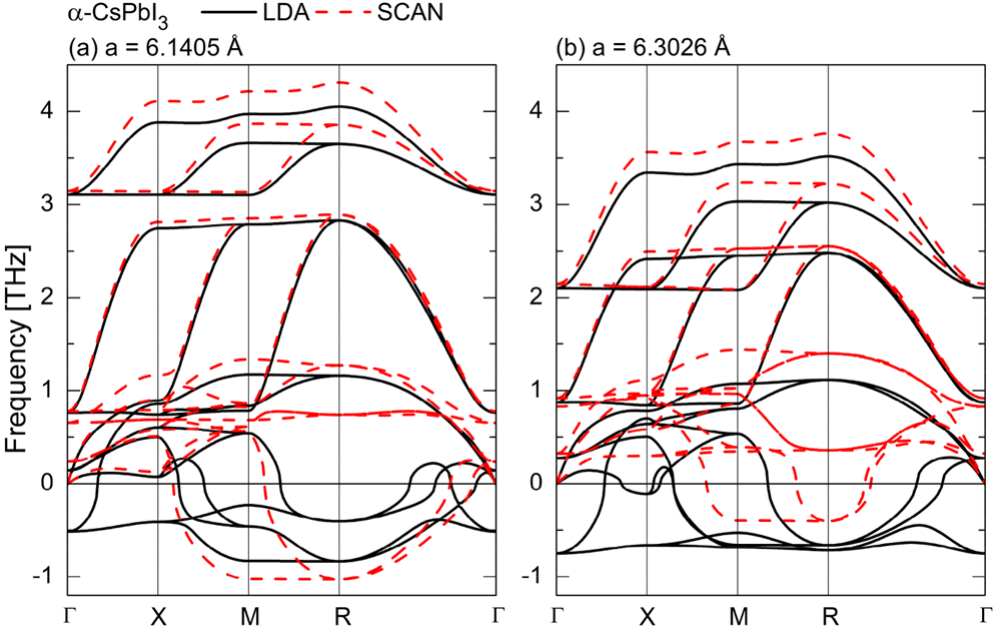
Phonon dispersion curves of the α-CsPbI_3_ calculated within both LDA and meta-GGA SCAN for (a) LDA and (b) SCAN volumes

**Figure 9:**
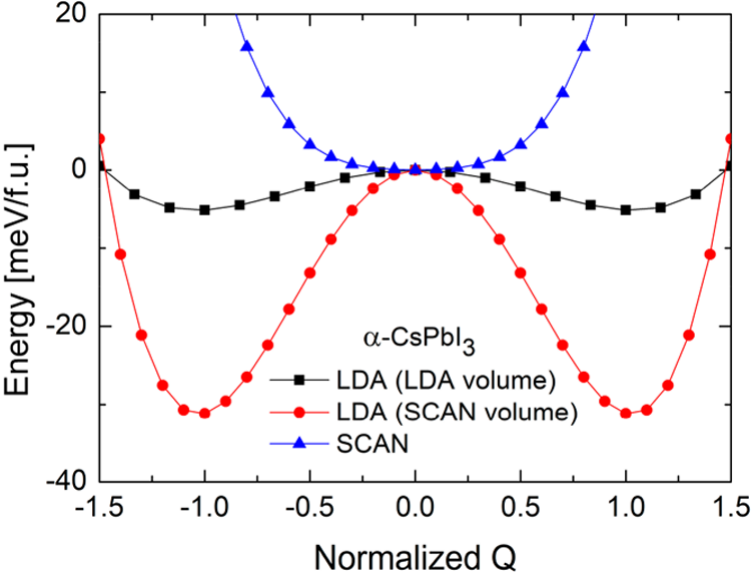
Potential energy surface along the eigenvector of the Γ-point soft mode as a function of normalized displacement *Q* calculated within the LDA for the α-CsPbI_3_ with the cell-volume obtained within LDA (black) and SCAN (red). The meta-GGA SCAN results for the same data are also presented (blue).

For further clarification of these discrepancies we calculated the electronic density of states of α-CsPbI_3_ within both LDA and SCAN functionals for the same volume. The results are presented in Figure 10. Except the slightly larger band gap within the meta-GGA SCAN (1.22 eV, compared to the value of 1.20 eV in the LDA) we did not find any difference between the electronic structure obtained from both approximations. The meta-GGA SCAN usually leads to the larger improvement of the band gap values compared to the LDA.^30,31^ This expectation results from the fact that SCAN better than the LDA and GGA cancels the self-interaction error (SIE) responsible for the well-known band gap problem in DFT. However, the SCAN itself is not free from the SIE. In ref (38), shown that the value of the band gap depends on the volume. In Figure 10 we see that the valence band consists mainly of the I(p) states with the small contribution from the Pb(s) states, whereas the conduction band is formed from the Pb(p) with the small contribution from I(s) states. As it was shown in ref (38), changing volume leads to the shortening of Pb–I bond lengths and, as a result, the interaction between Pb and I orbitals leads to the decreasing of the band gap. The values of band gaps for the optimized volumes within the LDA and SCAN are 0.96 and 1.22 eV, respectively. In fact, the SCAN functional improves the value of the band gap but this can be attributed to the better description of the structural parameters at the meta-GGA level.

**Figure 10:**
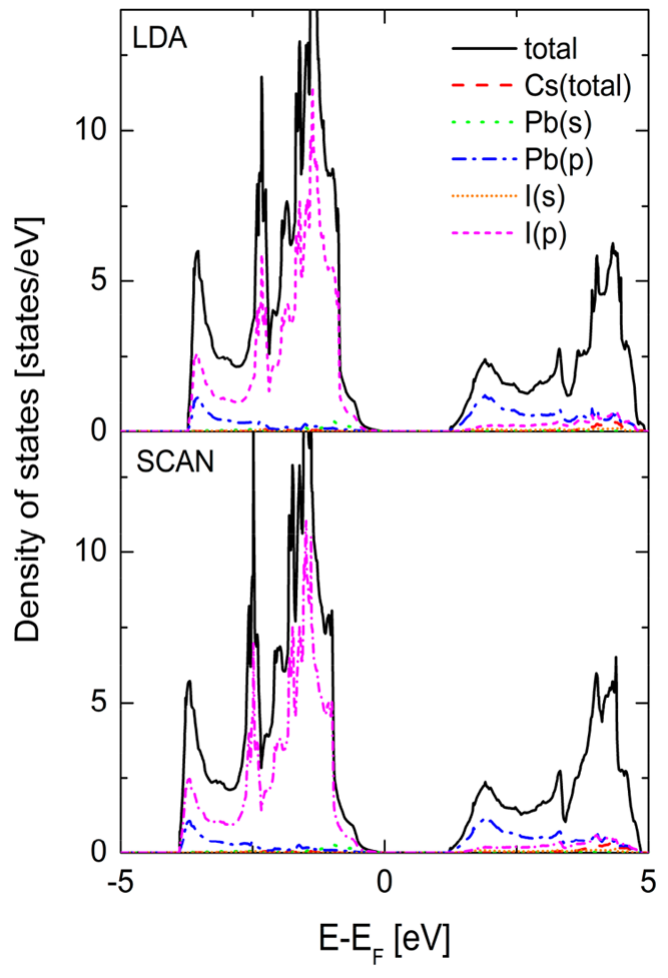
Total density of electronic states of the α-CsPbI_3_ phase calculated within both LDA (upper) and meta-GGA SCAN for the same volume (obtained within SCAN).

In summary, in this work we have performed calculations based on the density functional theory of structural, energetics, and vibrational properties of four known CsPbI_3_ polymorphs. We compared the results obtained within the local density approximation with those calculated within the meta-GGA SCAN. We found that meta-GGA SCAN led to the better agreement with the experimental data for the values of the structural parameters than the LDA. Moreover, within the meta-GGA SCAN we did not find the Γ-point soft mode in the phonon dispersion curve for both cubic and tetragonal phases of CsPbI_3_ responsible for the ferroelectric distortion. Such an instability is observed within the LDA. The SCAN result agrees with the lack of the observation of ferroelectric phases in CsPbI_3_. In addition, SCAN better than the LDA capture the temperature-dependent structural phase transition. To shed light on the difference results between the LDA and meta-GGA SCAN we also performed calculations of the phonon dispersion curves for different volumes within both approximations. The volume change does not affect qualitatively on the obtained results. We also compared the electronic density of states to confirm that the observed differences did not arise from the chemical bonds. Both LDA and meta-GGA SCAN lead to the similar densities of states. Above considerations lead to the conclusion that changes in the energy landscape as well as in the phonon dispersion behavior arise from the used exchange–correlation functional.

## Computational Methods

The density functional calculations were performed within the projector augmented wave (PAW) method^39,40^ as implemented in Vienna ab initio Simulation Package (VASP).^41,42^ The calculations were done within local density approximation (LDA)^43^ and strongly constrained and appropriately normed (SCAN) meta-generalized gradient approximation (meta-GGA).^28^ The additional test calculations were performed using generalized gradient approximation (GGA) using a parametrization proposed by Perdew, Burke, and Ernzerhof.^44^ The unit cell and atomic coordinates were optimized until the residual forces on constituent atoms become smaller than 10^–8^ eV/Å. The total energy convergence threshold of 10^–8^ eV was used. The kinetic energy cutoff for the plane-wave basis set was 800 eV. In ref (25) it was found that such a high value was necessary to converge phonon frequencies and eliminate artificial soft modes in the phonon dispersion curves. Our convergence tests for the LDA and SCAN meta-GGA functionals are presented in the Supporting Information (Figures S8–S11). The Brillouin-zone integrations were performed using 12 × 12 × 12, 8 × 8 × 9, 6 × 6 × 5, and 4 × 6 × 4 for the α-, β-, γ-, and δ-phases, respectively. Lattice dynamics calculations were performed using supercell approach as implemented in phonopy.^45−47^ The interatomic force constants were calculated in 2 × 2 × 2 supercells and 6 × 6 × 6, 4 × 4 × 5, 3 × 3 × 2, and 2 × 3 × 2 Γ-centered *k*-point grids for the α-, β-, γ-, and δ-phases, respectively. Figure 1 has been generated using the VESTA visualization package.^48^

## Supporting Information Available

The Helmholtz free energy difference for CsPbI_3_ polymorphs with respect to the CsI and PbI_2_ calculated within GGA. The comparison of the phonon dispersion curves obtained within the LDA and GGA was shown. The effect of the volume change on the phonon dispersion curves and the displacement of Cs and I atoms along the eigenvector of the Γ-point soft mode have been shown for the β-CsPbI_3_. The effect of the energy cut off on the phonon dispersion curves of CsPbI_3_ within the LDA and meta-GGA SCAN (PDF)
