# Supplementary material for: The Vibrational and Thermodynamic Properties of CsPbI3 Polymorphs: An Improved Description Based on the SCAN meta-GGA Functional
Source: J Phys Chem Lett. 2021 Jul 12;12(28):6613–21. doi: 10.1021/acs.jpclett.1c01798 (PMC8397336; doi:10.1021/acs.jpclett.1c01798)
Supplement: Supplementary file 1 — jz1c01798_si_001.pdf [file jz1c01798_si_001.pdf]

Supporting Information:

The vibrational and thermodynamic properties of $CsPbI_3$ polymorphs: an improved description based on the SCAN meta-GGA functional

*Jakub Kaczkowski**[a], *Iwona Płowaś-Korus*[a]

[a] Institute of Molecular Physics, Polish Academy of Sciences, M. Smoluchowskiego 17, 60-179 Poznań, Poland

Corresponding Author

*e-mail: kaczkowski@ifmpan.poznan.pl

1. The generalized gradient approximation (GGA) results Figs. S1-S5

2. The phonon dispersions curves and potential energy surface as a function of distortion for β-$CsPbI_3$ Figs. S6-S7

3. The effect of the energy cut off on the phonon dispersion curves of $CsPbI_3$ within the LDA and meta-GGA SCAN Figs. S8-S11

1. The generalized gradient approximation (GGA) results

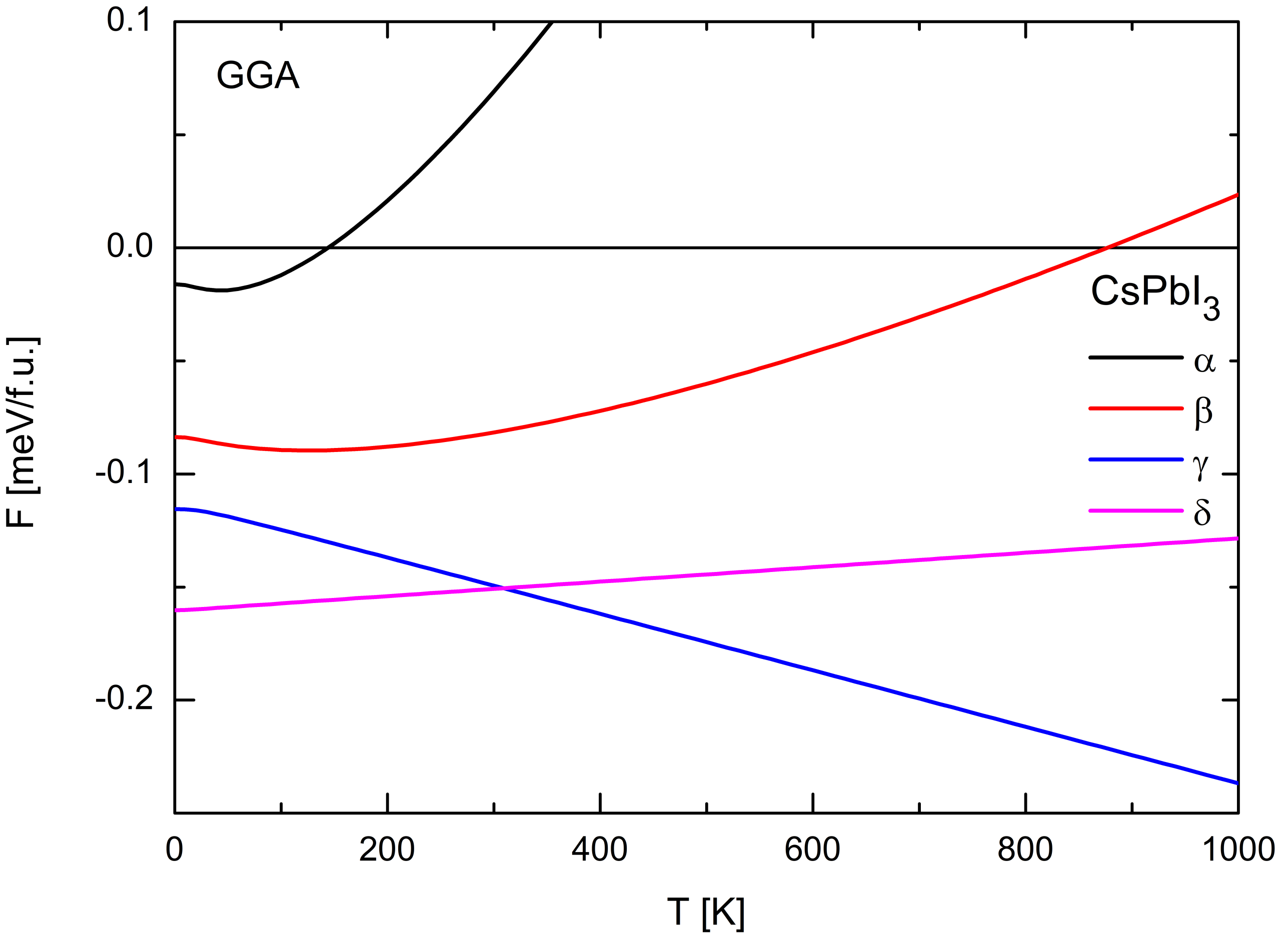

Figure S1. The Helmholtz free energy difference for $CsPbI_3$ polymorphs with respect to the CsI and PbI_2 calculated within the generalized gradient approximation (GGA).

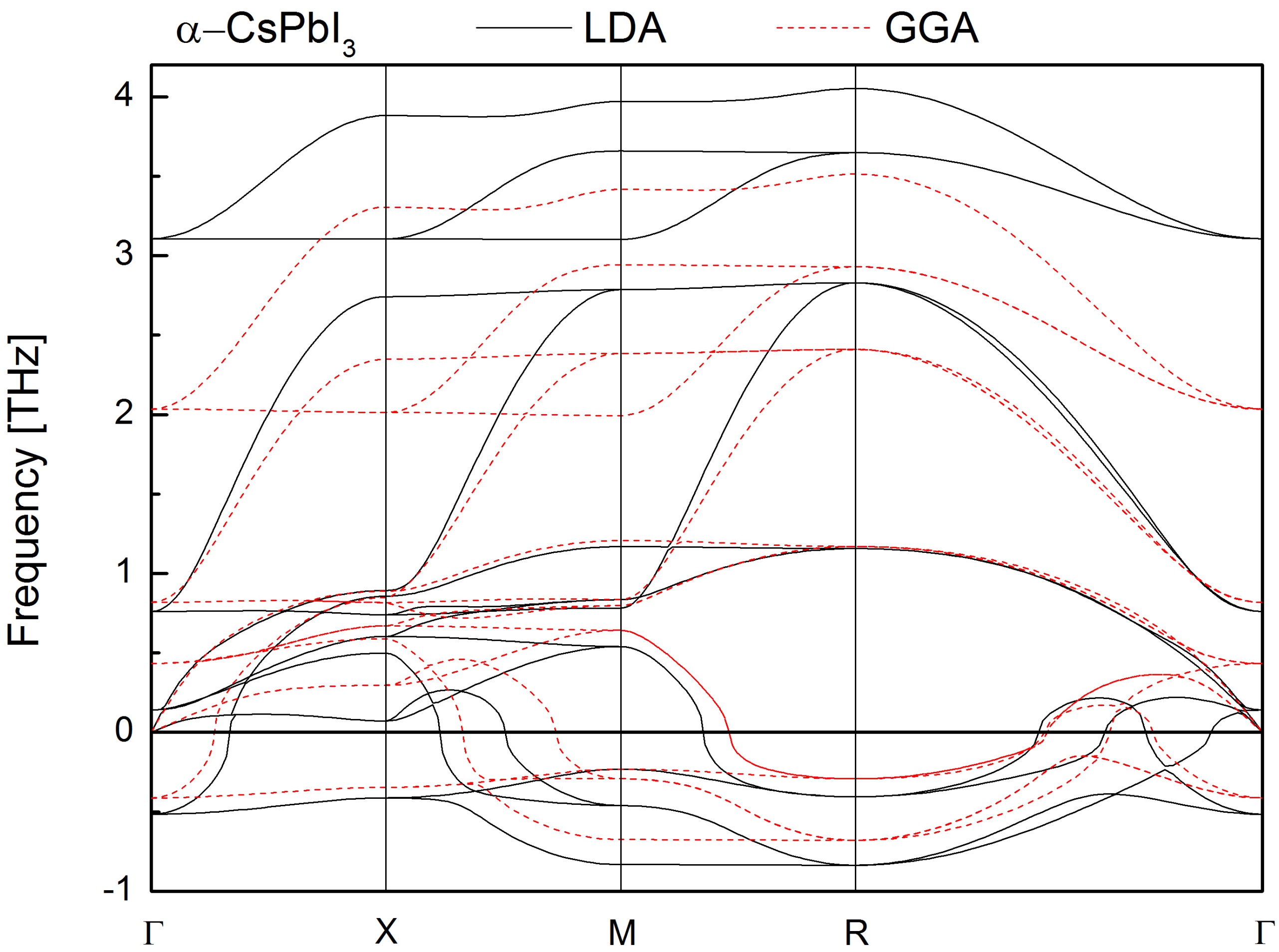

Figure S2. Phonon dispersion curves for α-$CsPbI_3$ calculated within the LDA and GGA at their predicted equilibrium volumes.

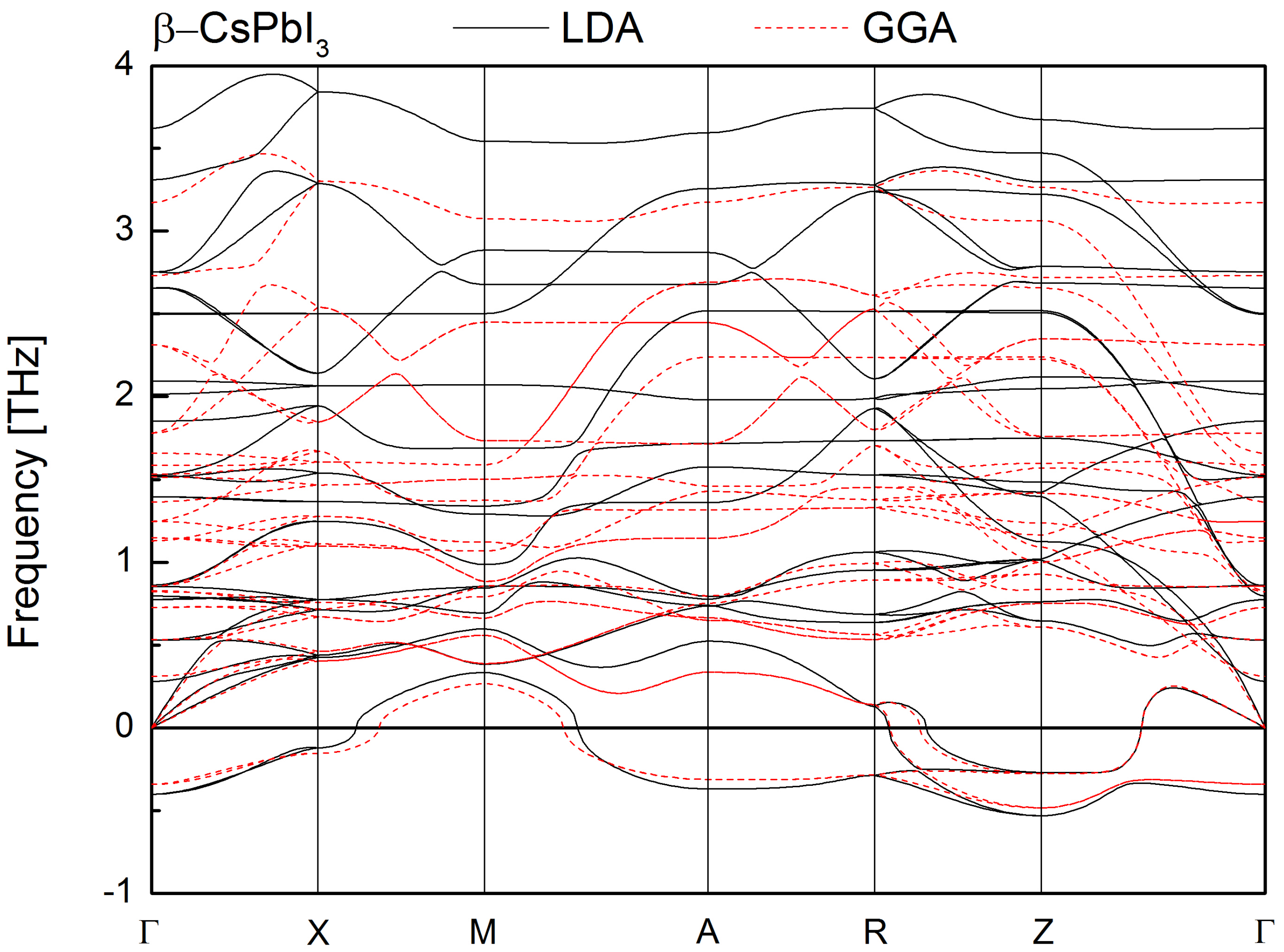

Figure S3. Phonon dispersion curves for β-$CsPbI_3$ calculated within the LDA and GGA at their predicted equilibrium volumes.

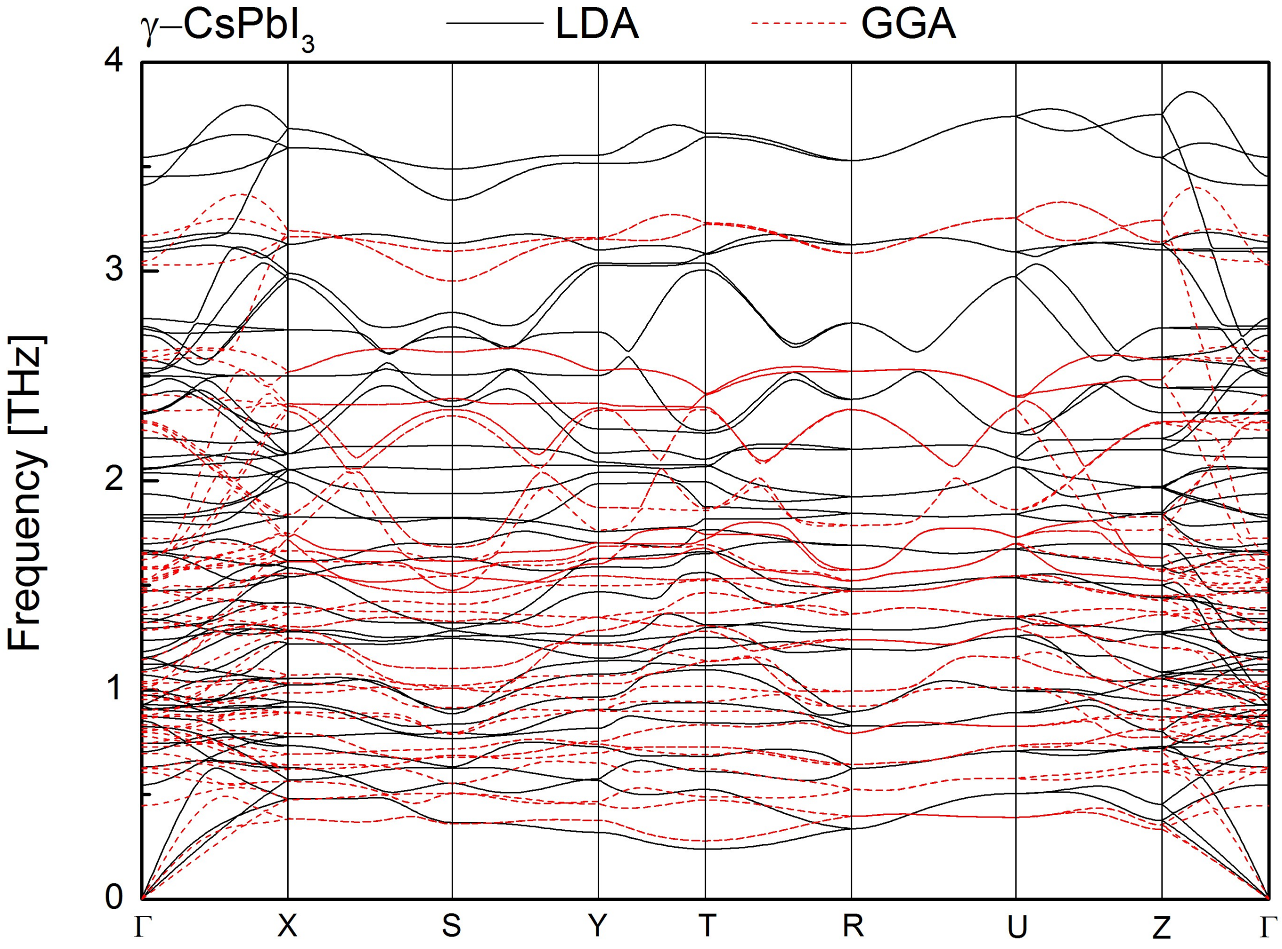

Figure S4. Phonon dispersion curves for γ-$CsPbI_3$ calculated within the LDA and GGA at their predicted equilibrium volumes.

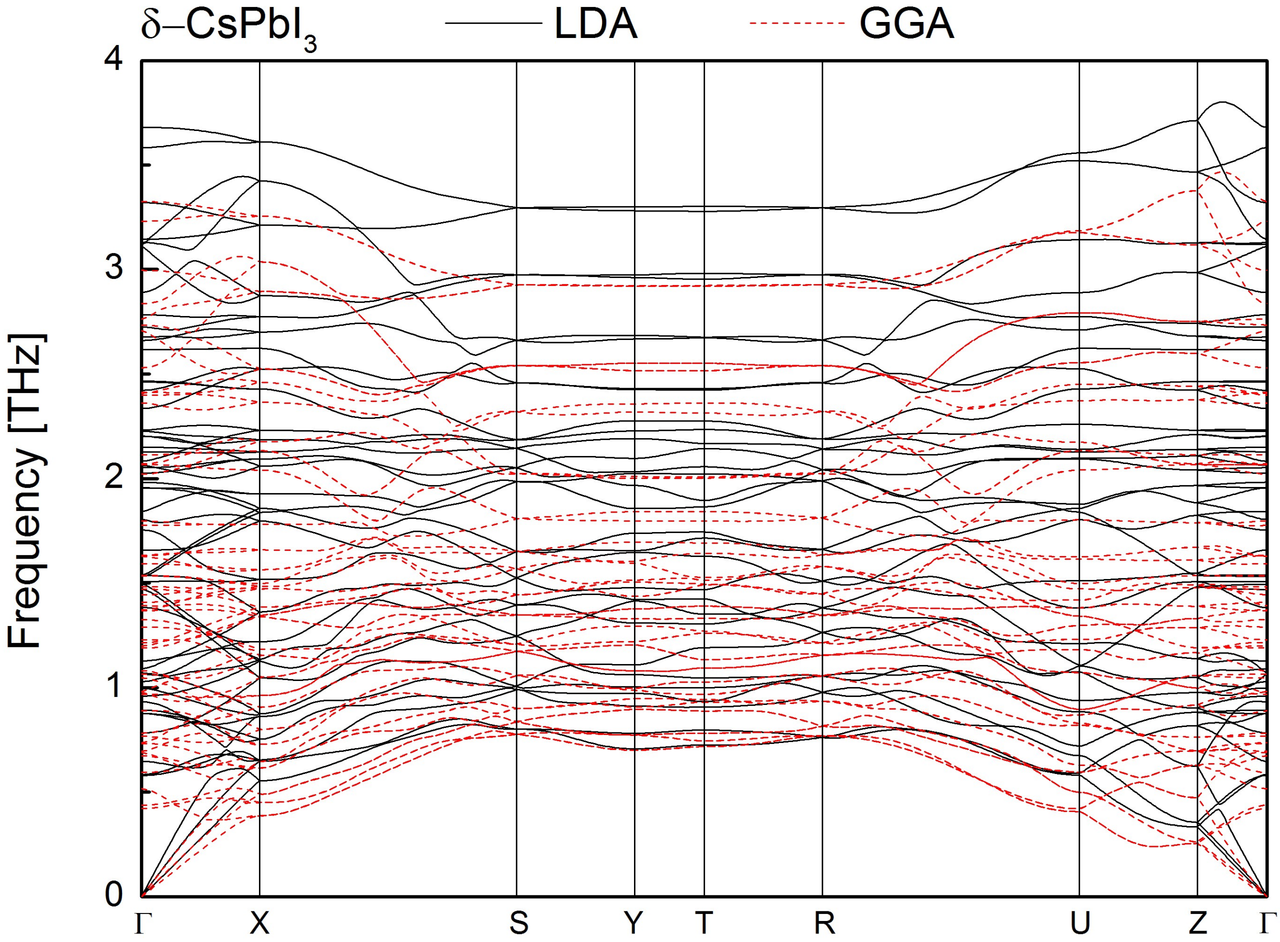

Figure S5. Phonon dispersion curves for δ-$CsPbI_3$ calculated within the LDA and GGA at their predicted equilibrium volumes.

2. The phonon dispersions curves and potential energy surface as a function of distortion for β-$CsPbI_3$

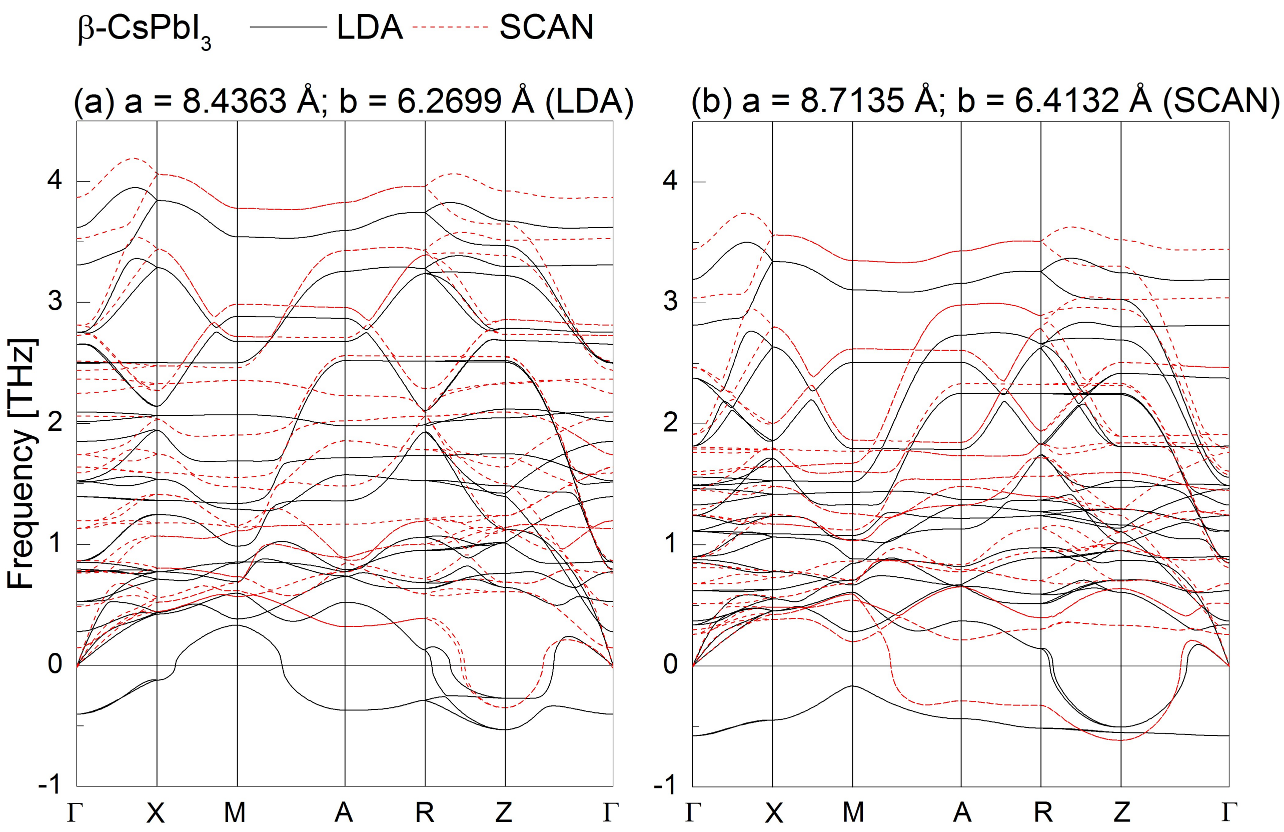

Figure S6. The phonon dispersion curves of the β-$CsPbI_3$ calculated within both LDA and meta-GGA SCAN for (a) LDA and (b) SCAN volumes.

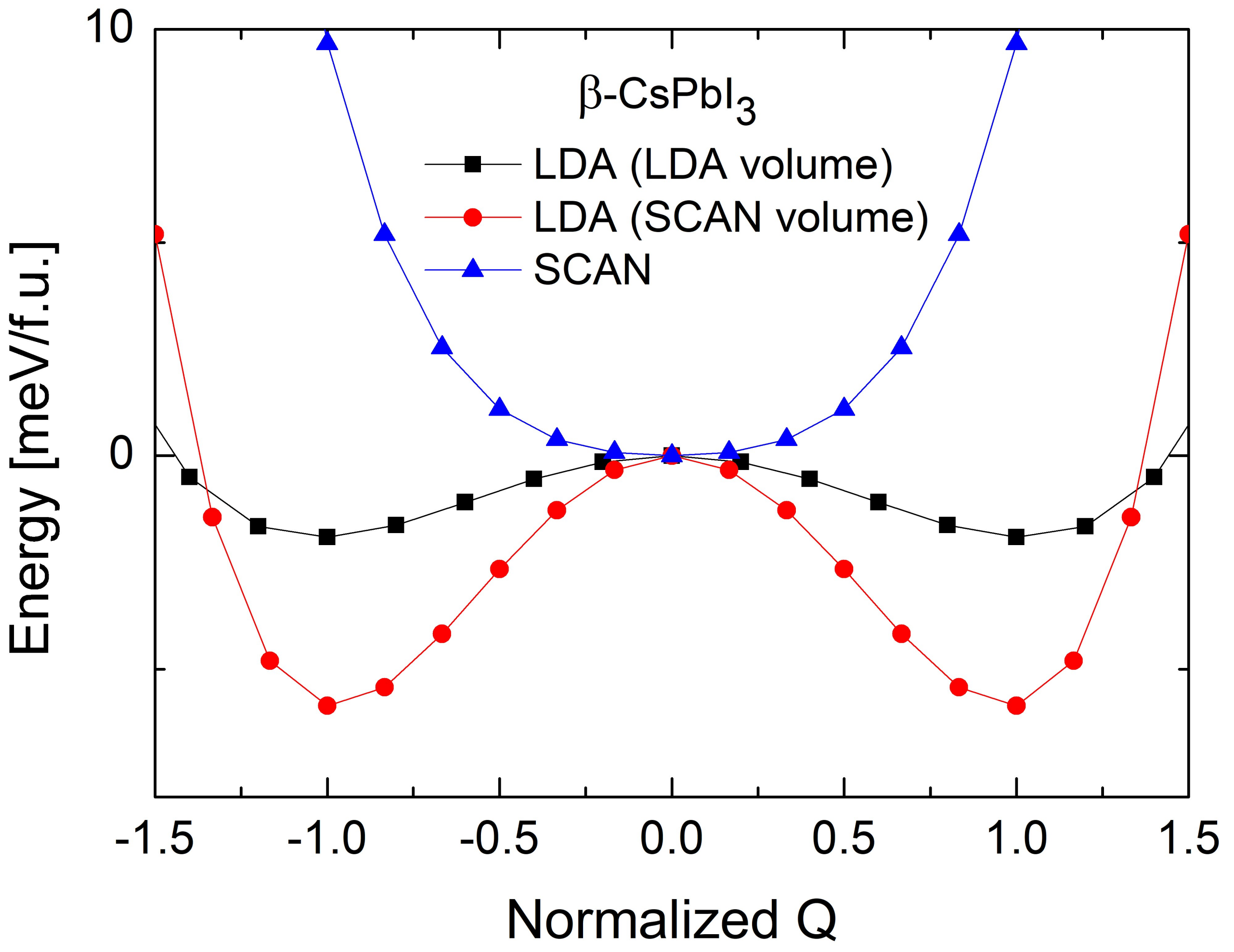

Figure S7. The potential energy surface along the eigenvector of the Γ-point soft mode as a function of the normalized displacement Q calculated within LDA for β-$CsPbI_3$ with the cell-volume obtained within LDA (black) and SCAN (red). The meta-GGA SCAN results for the same data are also presented (blue).

3. The effect of the energy cut off on the phonon dispersion curves of $CsPbI_3$ within the LDA and meta-GGA SCAN

In the most density functional theory (DFT) studies to ensure the reliability of the computational results the convergence tests for the cutoff energy for the plane wave basis set and the number of k-points are done with respect to the total energy. However, in Ref. [S1] it was shown that also for the phonon frequencies these tests are necessary to eliminate unphysical imaginary modes at the Γ point. In this Supporting Information we present the convergence tests of the cutoff energy for the phonon dispersions in the case of the cubic phase of $CsPbI_3$. In VASP the typical value of the energy cutoff for investigated compound is the highest value of ENMAX in the POTCAR file [S2]. In our case it was 220.32 eV. For the volume relaxation it is necessary to increase this value by 30% to avoid Pulay stress. Here, we choose two values of 600 eV and 800 eV for cutoff energy to test their effect on phonon dispersion. The results of our tests are presented in Figs. S8 and S9 for LDA and SCAN respectively. For the LDA we did not find any difference between phonon dispersion curves obtained for two values of the energy cutoff. For the meta-GGA SCAN functional there are visible difference at the lower frequencies of the spectra. We also checked the role of the semicore Pb-d states. The results are presented in Figure S10 for the meta-GGA SCAN. The effect of these states on the phonon spectra is negligible. Finally, in Figure S11 we presents the effect of the energy cutoff radius on the phonon spectra of the non-perovskite orthorhombic phase of $CsPbI_3$. For the value energy cutoff of 600 eV we observed soft modes near the Γ-point. Increasing cutoff from 600 eV to 800 eV leads to the significant decrease of these soft modes. To fully eliminate these modes further increase of the energy cutoff is probably necessary but this also leads to significant increase of the computational cost.

References

[S1] E. Lora da Silva, J. M. Skelton, S. C. Parker, A. Walsh, Phys. Rev. B 91 (2015) 144107
[S2] The VASP Manual https://cms.mpi.univie.ac.at

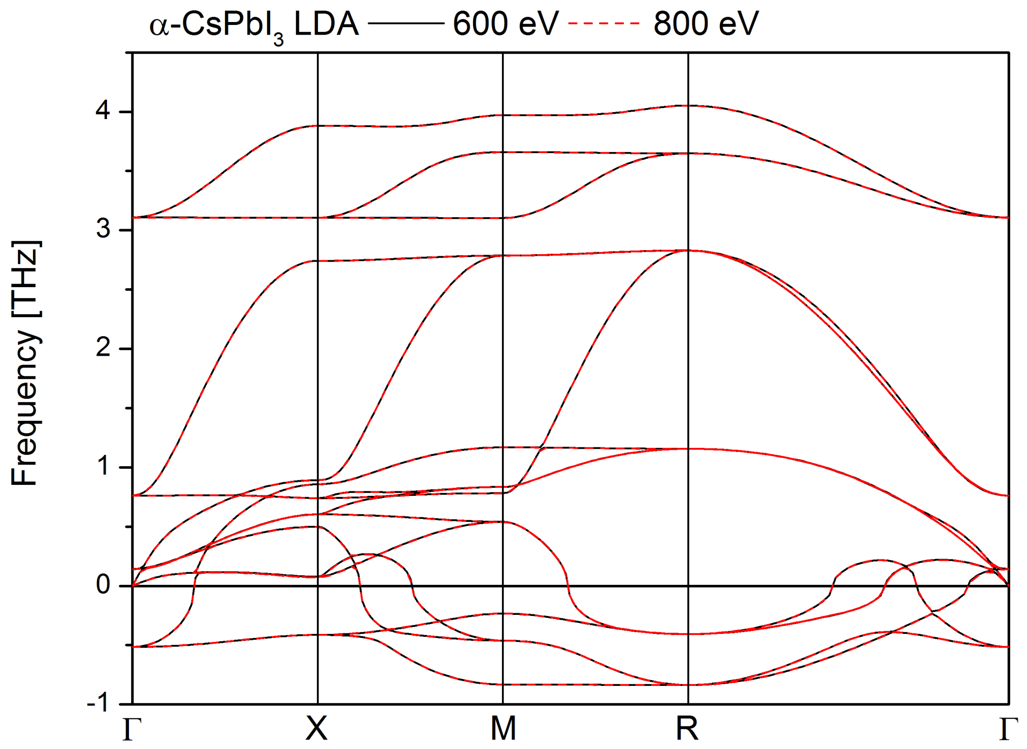

Figure S8. The phonon dispersion curves of the cubic $CsPbI_3$ phase calculated within the LDA for two values of the energy cutoff.

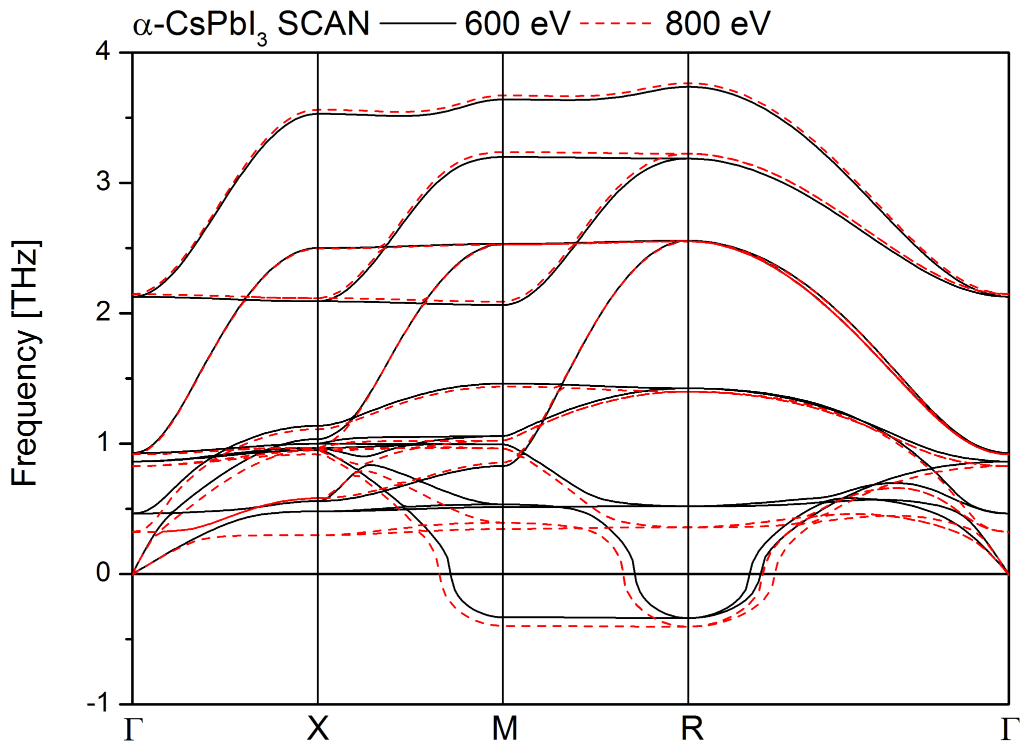

Figure S9. The phonon dispersion curves of the cubic $CsPbI_3$ phase calculated within the meta-GGA SCAN for two values of the energy cutoff.

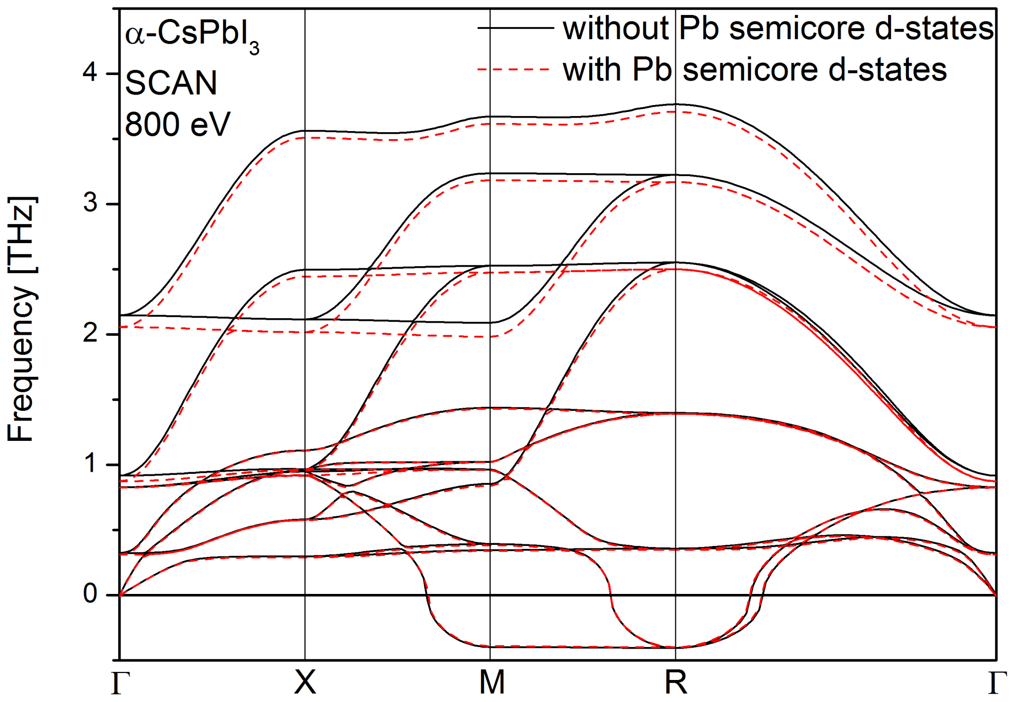

Figure S10. The phonon dispersion curves of the cubic $CsPbI_3$ phase calculated within the meta-GGA SCAN with (red dash) and without the Pb semicore d-states.

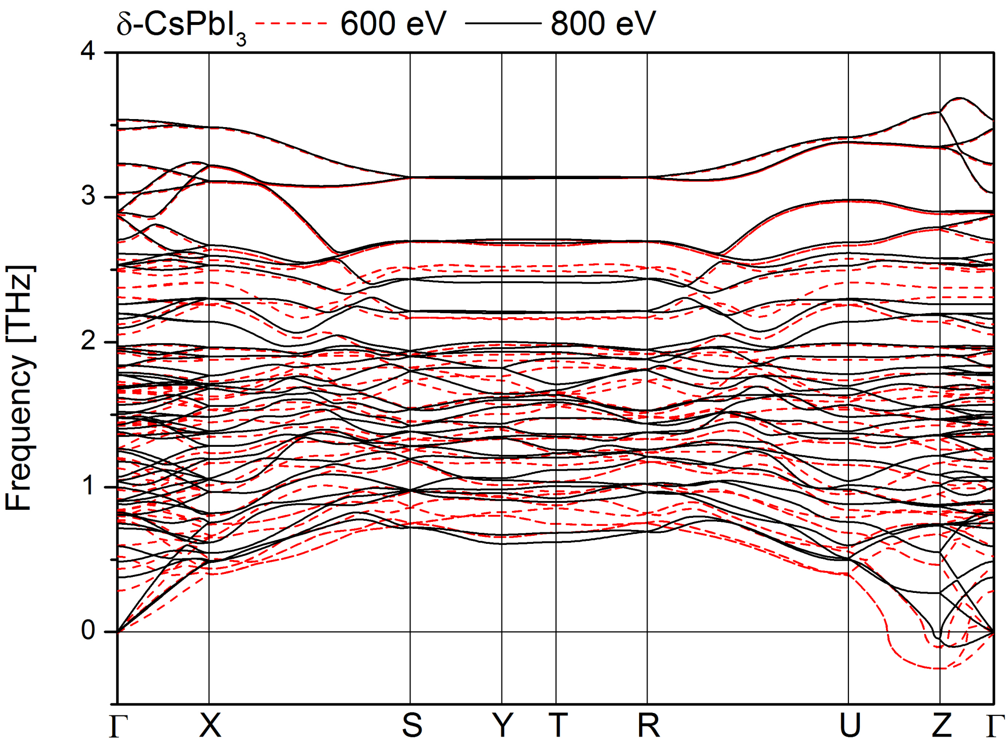

Figure S11. The phonon dispersion curves for the orthorhombic non-perovskite phase of $CsPbI_3$ calculated within meta-GGA SCAN functional for two different values of the energy cutoff.
